# Regulation of Adipose Tissue Stromal Cells Behaviors by Endogenic Oct4 Expression Control

**DOI:** 10.1371/journal.pone.0007166

**Published:** 2009-09-24

**Authors:** Jung Hwan Kim, Min Ki Jee, So Young Lee, Tae Hee Han, Bong Sun Kim, Kyung Sun Kang, Soo Kyung Kang

**Affiliations:** 1 Department of Veterinary Biotechnology, Seoul National University, Seoul, Republic of Korea; 2 Department of Veterinary Public Health, College of Veterinary Medicine, Seoul National University, Seoul, Republic of Korea; 3 Department of Pharmacology, and BK21 Program for Veterinary Science, Seoul National University, Seoul, Republic of Korea; Medical College of Georgia, United States of America

## Abstract

**Background:**

To clarify the role of the POU domain transcription factor Oct4 in Adipose Tissue Stromal Cells (ATSCs), we investigated the regulation of Oct4 expression and other embryonic genes in fully differentiated cells, in addition to identifying expression at the gene and protein levels. The ATSCs and several immature cells were routinely expressing Oct4 protein before and after differentiating into specific lineages.

**Methodology/Principal Findings and Conclusions:**

Here, we demonstrated the role of Oct4 in ATSCs on cell proliferation and differentiation. Exogenous Oct4 improves adult ATSCs cell proliferation and differentiation potencies through epigenetic reprogramming of stemness genes such as Oct4, Nanog, Sox2, and Rex1. Oct4 directly or indirectly induces ATSCs reprogramming along with the activation of JAK/STAT3 and ERK1/2. Exogenic Oct4 introduced a transdifferentiation priority into the neural lineage than mesodermal lineage. Global gene expression analysis results showed that Oct4 regulated target genes which could be characterized as differentially regulated genes such as pluripotency markers NANOG, SOX2, and KLF4 and markers of undifferentiated stem cells FOXD1, CDC2, and EPHB1. The negatively regulated genes included FAS, TNFR, COL6A1, JAM2, FOXQ1, FOXO1, NESTIN, SMAD3, SLIT3, DKK1, WNT5A, BMP1, and GLIS3 which are implicated in differentiation processes as well as a number of novel genes. Finally we have demonstrated the therapeutic utility of Oct4/ATSCs were introduced into the mouse traumatic brain, engrafted cells was more effectively induces regeneration activity with high therapeutic modality than that of control ATSCs. Engrafted Oct4/ATSCs efficiently migrated and transdifferentiated into action potential carrying, functionally neurons in the hippocampus and promoting the amelioration of lesion cavities.

## Introduction

A critical regulator of stem cell pluripotency, Oct4 (POU domain transcription factor, Pou5f1) is highly expressed in the early stage of mammalian embryo and in the inner cell mass of the blastocyst. Downregulation of Oct4 expression during trophoblast differentiation, the generation of mutant embryos, and conversion into exclusively trophoblast-like cells after Oct4 knockout revealed that the transcription factor, Oct4 was required to either establish or maintain pluripotency in the developmental embryo. For embryonic stem (ES) cell culture, even though the mechanisms of Oct4 involving cell fate regulation have been widely investigated, the postnatal role is still unclear.

Recent studies have shown Oct4 to be associated with the undifferentiated pluripotent state of stem cell populations derived from various adult tissues. To clarify the role of Oct4 in adult cells, we investigated the regulation of Oct4 expression and other embryonic genes in fully differentiated cells, while identifying that it was also expressed at the gene and protein levels in ATSCs. Along with Oct4, Sox2 also have been identified to be crucial for maintenance of the pluripotent state of ES cells [1], [2]. ES cells lose the capacity to maintain pluripotency upon knockdown of expression of these transcription factors by RNA interference. It has been demonstrated by chromatin immunoprecipitation studies that Oct4 and Sox2 bind to a few thousand regulatory sites in the ES cell genome [3], [4] and likely that many of these target genes play a modulating role in ES cell differentiation. In ES cells, Oct4 and Sox2 were shown to reciprocally regulate Pou5f1 and Sox2 transcription via the Oct4, Sox2 complex in ES cells [5]. Additionally, Oct4 and Sox2 positively regulate Nanog expression, revealing that the transcriptional regulation network was working to maintain the pluripotency of ES cells. Finally, Oct4's early function as a pluripotency maintaining transcription factor really acts as a gatekeeper, preventing differentiation along the trophoblast lineage [6]–[8]. A recent study about somatic cells reprogramming with specific genes showed that Oct4, Sox2, c-Myc and Klf4 played essential roles in stem cell pluripotency. These factors can induce reprogramming in somatic, mouse embryonic, and adult fibroblast cells to a pluripotent state. These four factors also have been seen to induce reprogramming in human dermal fibroblasts [9], [10]. Moreover, another group showed that Oct4, Sox2, Nanog and Lin28 were sufficient to establish pluripotent cells from human somatic cells [11]. On the other hand, several studies reported that Oct4 was detected in a variety of somatic tissue-derived cells, including bone marrow mesenchymal stem cells and subpopulation of the other adult cells [12], [13]. Several research groups have shown Oct4 to be dispensable for somatic stem cell function and their expression by several biochemical experiments. The majority of these studies concentrated on the identification and characterization of putative ‘pluripotent’ subpopulations of bone marrow-derived mesenchymal stem cells (MSCs).

Cell plasticity in stem cell biology specifies the ability of stem cells to differentiate into a variety of cell lineages; the term is also currently applied to the ability of a given cell type to reciprocally dedifferentiate, re-differentiate, and/or trans-differentiate in response to specific stimuli [14], [15]. Cell reprogramming signifies the withdrawal of cells from a given differentiated state into a stem cell-like state, a process that precedes re-entry into the cell cycle [13]. In this study, we utilized the stemness gene Oct4 to directly induce reprogramming of adipose tissue stromal cells (ATSC) into more primitive stem cells overexpressing stemness genes including Oct4, Sox2, and Nanog. Additionally, demethylation of the regulatory regions of stemness genes finally resulted in improved stem cell behavior of de-differentiated ATSC. Proliferation activity of reprogrammed ATSCs was induced by REX1, Oct4, and JAK/STAT3 directly or indirectly. Oct4/ATSCs showed increased migration activity that mediated by P38/JUNK and ERK phosphorylation. Moreover, the regenerative efficacy of Oct4/ATSC after engraftment into the traumatic injured brain was significantly restored their functions. Finally, our stem cell remodeling system may provide insight into the molecular mechanisms underlying ATSC proliferation and transdifferentiation. Also, these multipotent stem cells can potentially be harvested to provide a reservoir of primitive and autologous stem cells for use in a broad spectrum of regenerative cell-based disease therapy.

## Results

### Exogeneous Oct4 prominently improves ATSCs cell proliferation and differentiation potencies

**Figure 1:**
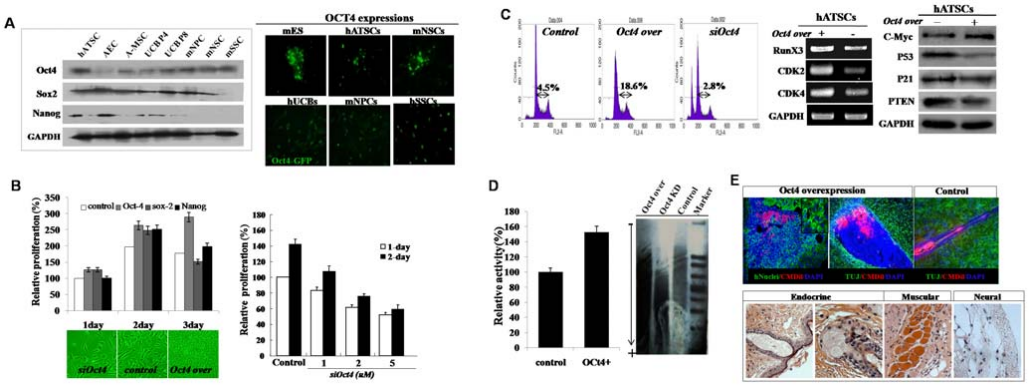
Oct4 actively enhances the proliferation of ATSC cells along with the overexpression of pluripotency related genes and telomerase activation. (A) Oct4 and several stemness genes expression in various kinds of adult stem cells. (B) The proliferation activity of Oct4 overexpressed ATSC cells. Oct4 was prominently enhances ATSC cells growth activity compare to Sox2 and Nanog genes. And knockdown of endogeneously expressing Oct4 prominently decrease cell proliferation activity and cell survival at a various siOCT4 concentration. (C) Cell cycle analysis in exogenic Oct4 expression and differential expression of checkpoint regulating gene expression in Oct4/ATSCs. For flow cytometric analysis, cells were cultured at densities that ensured exponential growth at the time of harvesting. Harvesting and processing protocols were used to detect DNA via flow cytometry with propidium iodide. The percentages of cells in the G0/G1, S, and G2/M phases of the cell cycle were determined using a DNA histogram fitting program. A minimum of 10^4^ events/samples was collected. (D) Telomerase activity and telomere length analysis. Oct4/ATSCs showed extended telomere length along with increased telomerase activity compare to control ATSCs. (E) Determination of differential expression of stemness and several pluripotency related genes by western blotting and real time RT-PCR. (F) Tumorigenic potency and three germ layer developing teratoma formation of Oct4/ATSCs.

To determine the expression of several stemness genes in tissue specific stem cells such as fetus-derived adult stem cells and mesenchymal stem cells, we performed western blot analysis and immunocytochemistry on cultured stem cells. As shown in Fig. 1A, several control adult stem cells highly expressed Oct4, Sox2, and Nanog. Oct4 expression in ATSC was higher than other stem cell species.

### Oct4 overexpression in ATSC as evidenced by various cell reprogramming behaviors via the expression of stemness genes

**Figure 2:**
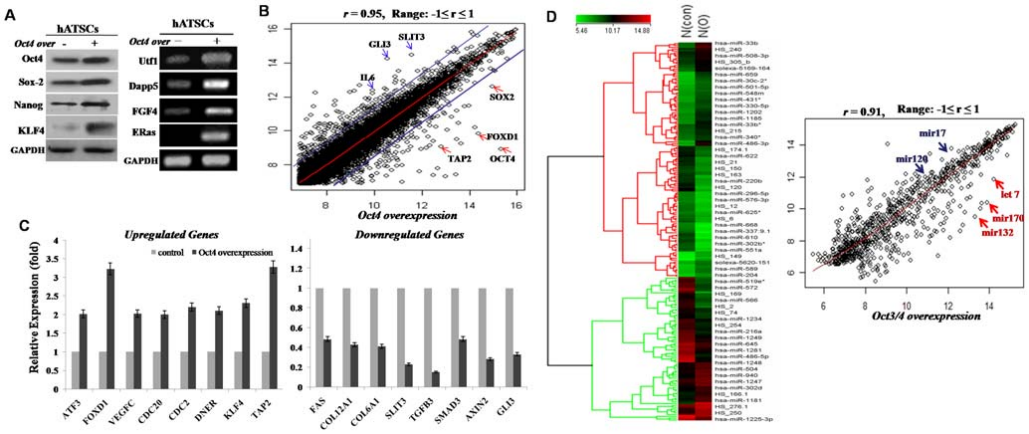
Exogenic Oct4 induced cell proliferation and survival related signal proteins expression in Oct4/ATSCs. (A) The involvement of JAK/STAT3, ERK1/2, MEK, and inhibition and activation resulted in prominent cell growth attenuation. Oct4 downregulation prominently induces inactivation of JAK/STAT3, AKT, MAPK, mTOR, and c-myc. When we treated with PI3K and ERK1/2 inhibitors, associated cell growth signal mediators and cell proliferation was decreased profoundly. For Western blotting, equal amounts of protein extracts were subjected to 10% SDS-PAGE analysis and transferred to nitrocellulose membranes. Optimally diluted antibodies were incubated with the membranes. The relative band intensities were determined using Quality-one 1-D Analysis software (B) Inhibition of Oct4 expression induced P38, SAPK/JUNK and mitochondria involving apoptotic ATSC death. Treatment of siOct4 actively induced apoptotic mediators such as caspases 8 and 9 and prominently increased apoptotic cell death along with highly increased Bax, PARP, and Cytochrome C expression or activation and Bcl2 downregulation.

The control ATSCs underwent a progressive reduction in proliferation potential, and finally underwent senescence after passage 25–30 (78–90 days in culture). As shown in Figure 2, after 3 days of *in vitro* culture, the Oct4/ATSCs expressed several stemness genes with extended cell growth. And Oct4/ATSCs overexpressed the oncogenic gene, c-myc with prominently extended the S phase in cell cycles (Fig. 1C). Oct4-overexpressing ATSCs induced a 1.5-fold increase in colony formation with increased synthetic DNA and telomerase activity along with slightly extended telomere lengths (Fig. 1D). The Oct4-overexpressed ATSC cells showed prominent effects on upregulation of a variety of proliferation-associated genes, including RUNX3, CDK2 and CDK4, and telomere reverse transcriptase (TERT; Fig. 1D). We evaluated the expression of Oct4 (POU5F1) to determine whether exogenic Oct4 induced the expression of early developmental genes KLF4, Sox-2, Rex-1, Utf1, Dapp5, FGF4, ERas, and Nanog in cultured ATSCs (Fig. 1E). Moreover, most of the Oct4 target genes were upregulated including Rex1, Nanog, and Sox2 (Fig. 1E). When Oct4/ATSCs were engrafted in the postnatal mouse brain, engrafted cells showed a strong tumorigenic potency *in vivo* (Fig. 1F). Engrafted Oct4/ATSCs presented highly neurogenic behavior in the fetal mouse brain 2 months after engraftment (Fig. 1F). Moreover, engrafted Oct4/ATSC cells in SCID/NOD mice formed the traditional teratoma morphology after 2–3 (n = 4) months. Oct4/ATSC-derived teratoma tissue developed into the three germs layers that tissues or organs such as endocrine gland tissue, muscle, and neural cells (Fig. 1F).

### Oct4 effectively induces ATSC reprogramming with JAK/STAT3 and ERK1/2 activation and Rex1 and Oct4 upregulations

In an effort to identify the potential signaling molecules involved in active cell proliferation following Oct4 overexpression, the total protein levels and phosphorylation status of ERK1/2 that well known cell proliferation mediator was assessed in the Oct4/ATSCs. Representative ES cells growth activating signal mediators, JAK/STAT3 and ERK1/2 phosphorylation was clearly upregulated at 12 hours (P<0.05) after exogenic Oct4 expression. Coinciding with Oct4/ATSCs proliferation, phosphorylated Akt was activated and was markedly increased after exposure to exogenic Oct4 expression in the Oct4/ATSCs (Fig. 2A). As following experimental results, Oct4/ATSCs proliferation was actively mediated by JAK/STAT3 and ERK1/2 phosphorylations (Fig. 2A). On the other hand, when we treated with PI3Kinase inhibitors, Akt and mTOR phosphorylations were decreased profoundly (Fig. 2A). As shown in Figure 2A, phosphorylated Akt was significantly upregulated after exogenic Oct4 expression. Oct4-induced ATSCs prominently activated AKT, MEK, MEKK and potentially raf proteins during cell proliferation, and also induced a profound reduction in ERK1/2 and p38 activation and cell growth activity following treatment with specific inhibitors (Fig. 2A). For further study on Oct4's role in the proliferation of ATSCs, the ATSC were transfected with Oct4 siRNA prior to and after induction of exogenic Oct4 expression. Akt, PI3K, ERK1/2, JAK, and STAT3 were also downregulated with the attenuation of cell proliferation by Oct4 inhibition (Fig. 2A). Moreover, the exogenic Oct4 induced cell survival after H_2_O_2_ exposure was shown to be regulated by the downregulation of p38/JUNK phosphorylation along with the survival signal mediator Bcl2 overexpression and apoptotic signal proteins downregulation such as Bax, Cytochrome C, and c-PARP along with Caspase3, 8, 9 inactivations. And P38/JUNK-mediated apoptotic cell death was prevented by inhibition of their phosphorylation (Fig. 2B). Following the Oct4 knockdown experiment, Oct4 was shown to be actively involved in cell proliferation and survival against external chemical stresses after exogenic Oct4 induction (Fig. 2B).

### Defective DNA methylation of stemness genes in exogenic Oct4 induced ATSCs

**Figure 3:**
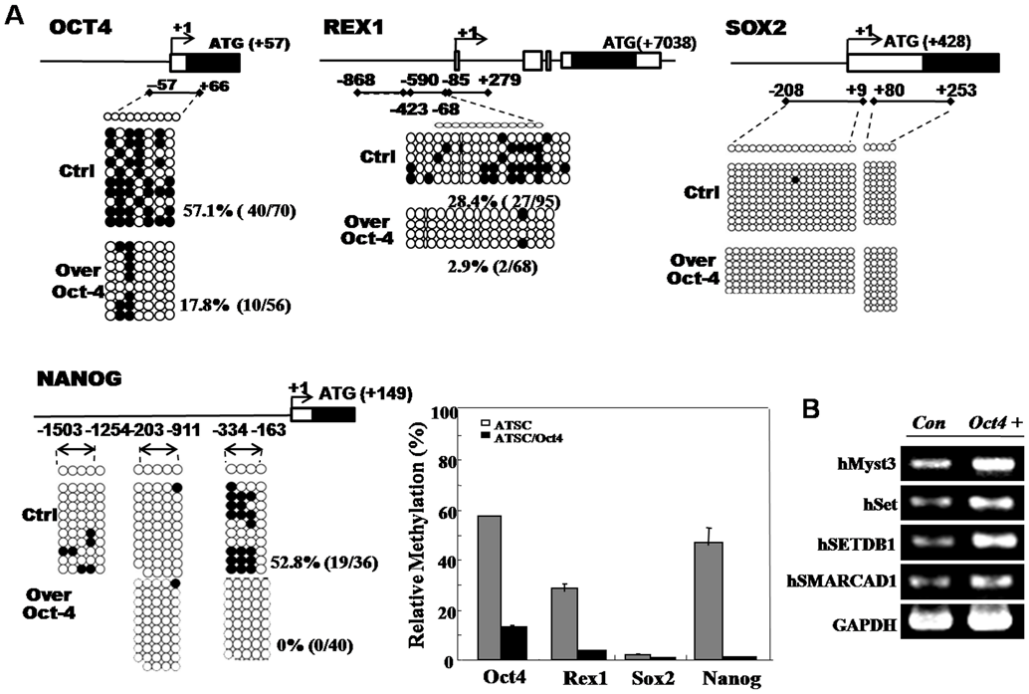
Evaluation of epigenetic modifications through methylation analysis of the promoters regions of Oct4, Rex1, Sox2, and Nanog genes. (A) Evaluation of epigenetic modifications through methylation analysis on promoter regions of Oct4, Sox2, Rex1, and Nanog through bisulfite modification and sequencing of genomic DNA. The related method was explained in supplementary materials and methods section. (B) Differential expression pattern of epigenetic reprogramming related genes such as hMyst3, hSET, hSETDB 1, and hSMARCAD 1 before and after Oct4 overexpression was detected by RT-PCR.

The analysis of gene expression levels indicated that <4% of the total genes was expressed greater than 2.2-fold for different levels in ATSC and Oct4/ATSCs. A comparison of the expression of these showed that the cell proliferation-associated genes were prominently upregulated in Oct4/ATSCs (Table S1, 39%). In an effort to determine whether Oct4 overexpression was capable of eliciting epigenetic modifications on exogenous chromatin templates of Oct4 and the other controllable stemnesses, Sox2, Nanog and Rex1. We evaluated changes in DNA methylation pattern in the stemness genes, Oct4, Sox2, Rex1, and Nanog promoter regions. We also conducted a bisulfate sequencing analysis in order to establish the 5′-3′ CpG methylation profiles across each test gene proximal promoter, the proximal enhancer, and the early transcription start site (TSS). In the case of Rex1, converting the potentially methylated CpG dinucleotides occurred within −590 to −85 nucleotides relative to the TSS (Fig. 3). For the Oct3/4 promoter encompassing the CpGs, −57 to +66 nucleotides relative to the TSS was analyzed (Fig. 3). The proximal Sox2 and the TSS regions did not significantly alter the methylation. We also assessed three regions of the Nanog promoter, encompassing a total of 3 CpGs within −1503 to −163 nucleotides relative to the TSS. The Rex1 region assessed was methylated in the control ATSCs and was meaningfully demethylated (Fig. 3). These Oct4 methylation patterns was prominently downregulated in the Oct/4/ATSCs compared to the control ATSCs (Fig. 3). Finally, exogenic Oct4 overexpression effectively induces Sox2, Nanog, and Rex1 demethylation. Our study has also provided some additional observations regarding nuclear remodeling, including the acetylation of histone H3 (Fig. 2A).

### Global gene expression analysis and transcriptional regulation

**Figure 4:**
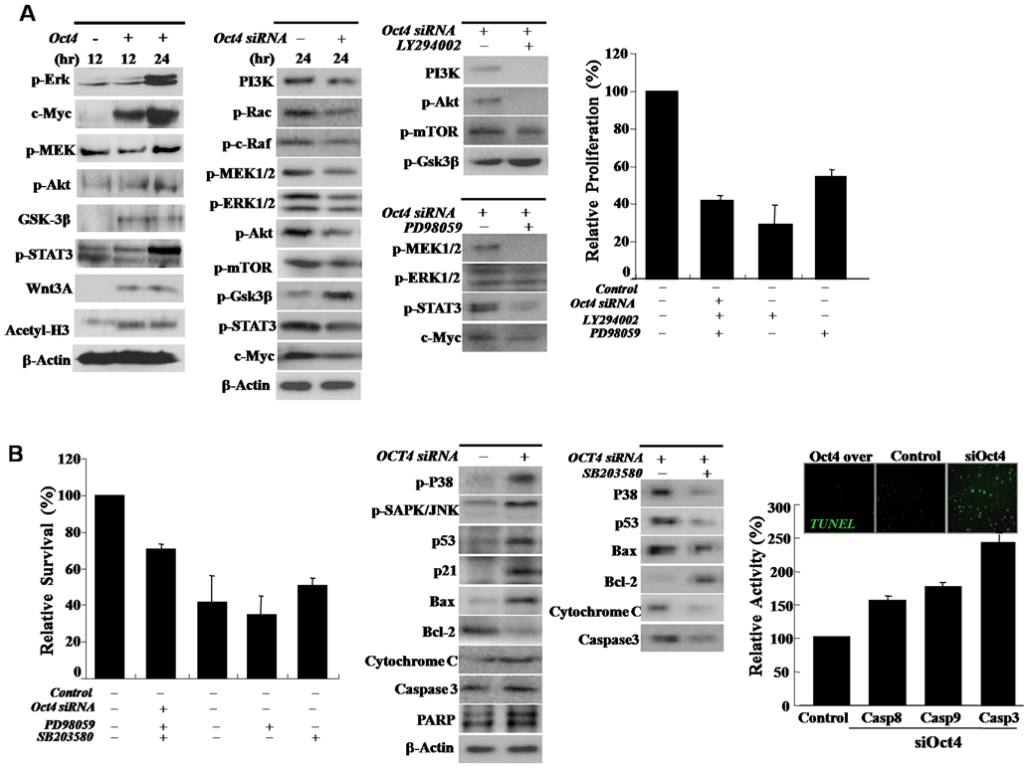
Differentially expressed mRNA and miRNA profiles in Oct4/ATSCs. (A) Global gene expression patterns were compared between control ATSCs and Oct4/ATSCs with DNA microarray. Blue lines indicated that twofold changes in gene expression levels between the paired cell types. Verification of differentially expressed genes in Oct4/ATSC cells (lower panel). (B) The heat map of the different expressed genes between control (Con) and Oct4/ATSCs (O). Positions of several genes including Sox2, FOXD1, and Oct4 and miRNA in scatter plots are indicated. Correlation Coefficiency value of the expression of miRNA in Oct4/ATSCs and control ATSCs was about 0.91. Several miRNA including mir132, mir170, and let7 were highly upregulated after Oct4 overexpression in ATSCs. In contrast of that, mir17 and mir120 was dramatically downregulated in Oct4/ATSCs. The gene expression levels were normalized using the RMA algorithm.

To identify genes with altered expression levels before and after Oct4 overexpression in cultured ATSCs, transcriptional profiles were generated by using a cDNA microarray consisting of 20,000 resequenced and annotated clones. We compared the 48 hour replicate after Oct4 overexpression with EGFP knockdown using statistical tests for differential expression. Among differentially expressing genes, almost 7,000 (75%) genes were overexpressed, and another 4,600 (25%) genes were downregulated. Both sets of markers genes were at a 0.5 significance level. These differentially regulated marker genes could be characterized regulated target genes by Oct4 such as pluripotency markers NANOG, SOX2, and KLF4 and markers of undifferentiated stem cells FOXD1, CDC2, and EPHB1. The negatively regulated genes included FAS, TNFR, COL6A1, JAM2, FOXQ1, FOXO1, NESTIN, SMAD3, SLIT3, DKK1, WNT5A, BMP1, and GLIS3 which were implicated in differentiation processes as well as a number of novel genes (Table S1). To confirm the negatively regulated downstream targets of Oct4, the profile analysis was repeated for BMP4. The gene encoding BMP4 is also highly expressed in the human trophectoderm [16] and has been shown to promote human embryonic stem cell differentiation into trophoblasts [17]. Included in this set were genes implicated in ESC differentiation, such as the transcription factors ID3, TBX18, GATA6, and HLX1 [18], and signal transduction pathways crucial for the maintenance of pluripotency, such as WNT (DKK1, FRZB, FZD2), transforming growth factor (TGF-β), and FGF (FGF8); and cytoskeletal and extracellular matrix associated genes such as COL4A1, LOXL4, and CLDN4, which encodes a tight junction protein crucial for trophectoderm formation. Differential expression of selected genes was verified independently using real time PCR and prepared from Oct4 overexpressed ATSCs or control ATSCs (Fig. 4). Confirmation of the differential expression of genes was verified and shown in Figure 4. This revealed that the upregulation of key pluripotency-controlling genes, OCT4, SOX2, and NANOG, and ES-associated genes KLF4, CEBP, FOXD1, HMGA1, and DNER. Differentially regulated genes are listed in Table S1.

### Regulatory miRNA expression in Oct4/ATSCs

We evaluated differentially expressing mature miRNAs in Oct4 overexpressed ATSCs. After global normalization of the raw data, 880 differentially expressed miRNAs were identified between control and Oct4/ATSCs using a cut off p value of <0.01 to narrow down the candidates. Among these miRNAs, 208 were significantly up- or downregulated in Oct4 overexpressed ATSCs (p<0.00032). After global normalization, hierarchical clustering analysis based on differentially expressed miRNAs generated a tree with clear distinction between control and Oct4 ATSCs (Fig. 4B). MiRNAs highly differentially expressed in Oct4/ATSCs were as follows; let 7, mir 170, mir 132 (upregulated in Oct4/ATSCs) and let 17 and let 120 (downregulated in Oct4/ATSCs). Correlation of the coefficiency of total miRNA expression in both of cell groups showed *r* = 0.91 (Range: −1≤r≤1) (Fig. 4B). To validate the results of our miRNA array analysis, 6 miRNAs were selected as up- and down-regulated miRNAs in Oct4 transfected ATSC cells by real time RT-PCR.

### Exogenic Oct4 introduces neural transdifferentiation priority but prominently attenuates mesodermal differentiation

**Figure 5:**
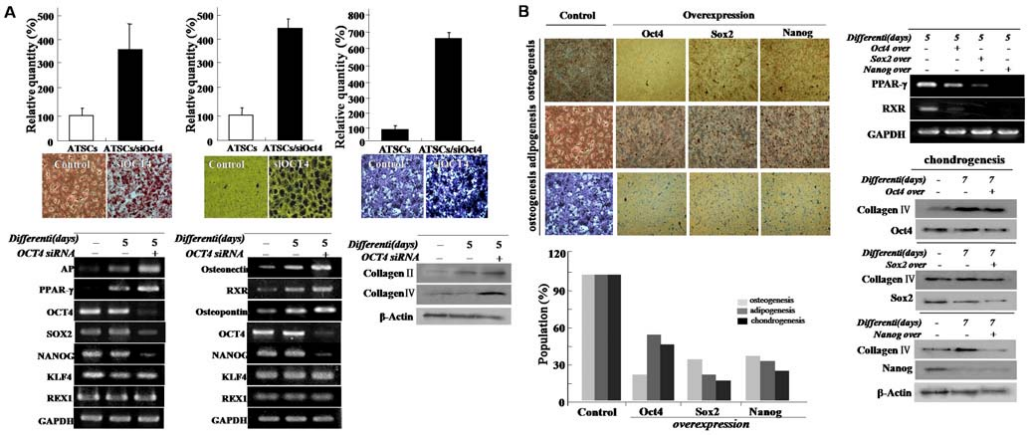
Effect of exogenic Oct4 and several stemness gene expressions on adipogenic, osteogenic, and chondrogenic differentiation potencies. (A) Oct4 knockdown actively induces adipogenic, osteogenic, and chondrogenic differentiation. Differentiation potency was evaluated by fat, bone, and chondrocyte staining and lineage specific gene expression pattern by RT-PCR or western blotting using tissue specific markers. (B) Exogenic stemness genes expression affect on mesodermal differentiation potency. After differentiation induction of Oct4/ATSCs and control ATSCs, the number of positive clones was quantified after fat (Oil Red O), bone (Van Kossa), and chondrocyte staining (Van Gieson) and data presented relative density of positive population (%). (C) Effect of stemness genes overexpression on differentiation of ATSCs into bone, fat, and chondrocyte and their lineage specific gene expression analysis after differentiation induction.

**Figure 6:**
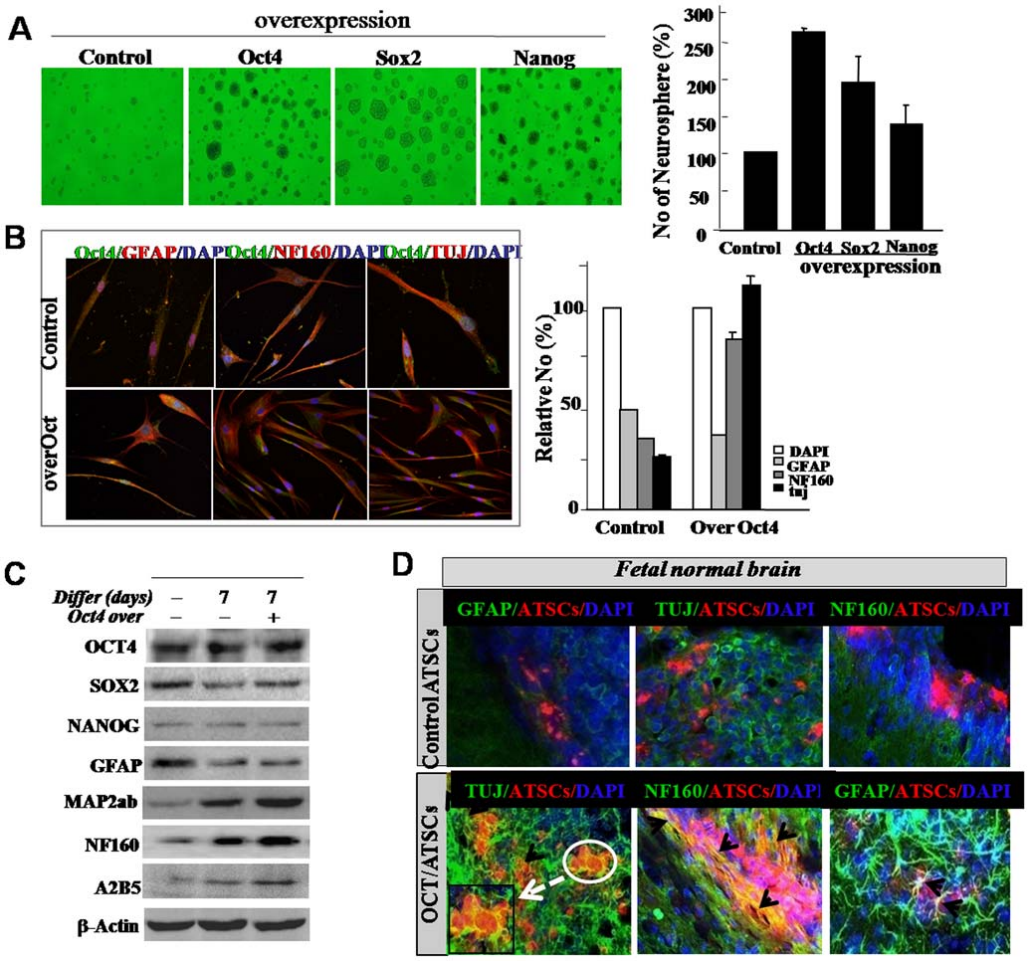
The transdifferentiation potency of Oct4/ATSCs to a neural lineage *in vitro* and *in vivo*. (A) Overexpression of several stemness genes enhances increased neurosphere formation. Neurospheres were counted after genes transfected ATSCs suspended in NB media culture and compared to the number of neurospheres derived from control and stemness overexpressed ATSCs. (B) Comparison of neural differentiation efficiency after neural differentiation induction between control ATSCs and Oct4/ATSCs. After neural differentiation, differentiation efficiency was evaluated by immunocytochemistry using anti-Tuj, anti-NF160, and anti-GFAP antibody and fluorescence-tagged secondary antibodies and we also analyzed stemness genes and neural specific gene expression pattern through (C) western blotting analysis. (D) Evaluation of *in vivo* integration and neurogenesis of engrafted Oct4/ATSCs in postnatal mouse brain using the immunohistochemistry method. Arrows indicate differentiated Oct4/ATSCs into MAP2ab-positive neurons.

ATSCs have been identified as skeletal tissue progenitors, and differentiate into osteoblast-like cells in cultures supplemented with ascorbic acid with a glucocorticoid source. Calcium and lipid droplets begin to accumulate in ATSCs following 2–4 weeks of induction in osteogenic and adipogenic differentiation media. We studied the effect of Oct4 overexpression on mesodermal lineage differentiation into fat, bone, and chondrocyte of ATSCs. Oct4 knockdown ATSCs were shown to accumulate significant quantities of calcium and lipid droplets, and differences were apparent in the efficiency of nodule and lipid droplet formation between the naive and siOct4/ATSC. As shown in Figure 5A, up to three times as many lipid droplets and nodules were detected in siOct4/ATSCs compared to the control. After culturing siOct4/ATSCs (passage 5) in osteogenic differentiation media, cells were stained for calcium deposits. These results were generally consistent with what has been observed in conjunction with the overexpression of adipogenesis- and osteogenesis-related transcription factors, including RXR, osteonectin, AP, and PPAR-gamma, after Oct4 mediated cell reprogramming (Fig. 5A). Moreover, when mesodermal differentiation was induced of Oct/4, Sox2, and Nanog overexpressed ATSCs, the differentiation efficiencies of each gene was prominently decreased along with each lineage specific (Fig. 5A and 5B). To determine the transdifferentiation activity of the Oct4/ATSC, the neurogenic potency was evaluated through transdifferentiation into a neural lineage. After neural induction in differentiated Oct4/ATSCs, we observed an extreme upregulation of TuJ and MAP2ab and low levels of Nestin expression in Oct4/ATSCs after neural differentiation and also neurosphere formation efficiency was higher than control ATSCs (Fig. 6A). When we engrafted Oct4/ATSCs in the fetal mouse brain, the engrafted cells effectively transdifferentiated into TuJ- and NF160-positive neurons in contrast of that of control ATSCs 5 weeks after engraftment (Fig. 6D). A large population of differentiated Oct4/ATSCs showed phenotypic characteristics of astrocytes (GFAP), and neurons (MAP2ab and NF160 [approximately 45–60% of the total population]; Fig. 6D). The siOct4/ATSCs did not appear to have neurogenic morphology and neural gene expression 7–10 days after neural differentiation induction (data not showed).

### Exogenic Oct4 induced improved regeneration activity in brain trauma with high therapeutic modality

**Figure 7:**
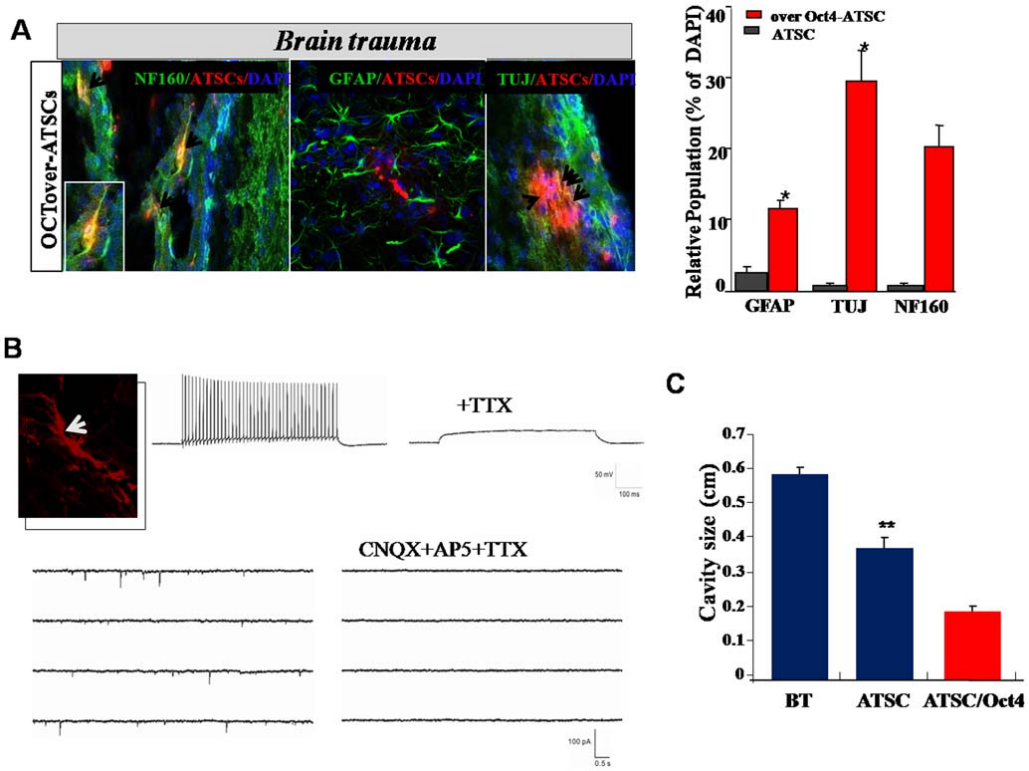
The therapeutic efficacy of engrafted Oct4/ATSCs in a mouse model of a traumatic brain injury. (A) High neuroregenerative potency of Oct4/ATSCs in injured lesion of traumatic mouse brain. The arrows indicate engrafted and functionally transdifferentiated neurons in the hippocampus. (B) Electrophysiological properties of transdifferentiated neuron from engrafted Oct4/ATSCs from a lesion of traumatic injured brain slice. Whole-cell patch-clamp recordings of engrafted Oct4/ATSCs in traumatic brain slice. The presence of the ionic currents was first investigated by applying voltage ramps between 120 and 120 mV to the cells patch clamped using K-based pipette solution. ATSCs control engrafted brain slice does not display inward current, whereas Oct4/ATSCs engrafted brain slice actively produced a current. This current was approximately 75% blocked by 30 nM tetrodotoxin (TTX), although 300 nM TTX blocked it completely. Oct4/ATSCs engrafted brain slice express voltage-gated Na channels. Voltage-clamp recording at70 mV in extracellular solution containing 3 mM Mg2. Traces show spontaneous slow and fast currents that indicate that this transplanted Oct4/ATSCs receives synaptic contacts from host cells. This current was approximately 98% blocked by CNQX AP5 solution. (C) The Oct4/ATSCs engrafted traumatic brain demonstrated that there was a decrease in the lesion cavity size.

To determine the regenerative activity of Oct4/ATSCs for *in vivo* brain trauma, we evaluated the neurogenic potency through transdifferentiation of the Oct4/ATSCs. After neural induction, Oct4/ATSCs differentiated into neural cells expressed higher levels of TuJ than in differentiated control ATSCs (Fig. 7A). Extremely down-regulated Nestin expression was observed in Oct4-induced neural cells after induction (data not showed). Moreover, in *in vivo* lesion sites of injured brains, TuJ-positive neuron subpopulations generally survived or actively trans-differentiated into neurons after Oct4/ATSC engraftment (Fig. 7A). Immunohistochemical analyses of brains with traumatic injuries revealed that a large population of differentiated neural cells from Oct4/ATSCs displayed morphological and phenotypical characteristics of neurons (TuJ; approximately 35–40% of the total population) with highly improved rotor-rod activity (data not showed).

### Functional analysis of the neuronal properties of Oct4/ATSCs *in vitro*

To confirm that adult human Oct4/ATSCs were differentiating into functionally active neurons, electrophysiology was performed on engrafted traumatic brain slices (Fig. 7). The presence of the ionic currents was first investigated by applying voltage ramps between 120 and 120 mV to the patch clamped cells using a K-based pipette solution. Engrafted brain slices with control ATSCs did not display an inward current, whereas the brain slices with Oct4/ATSCs actively produced a current (Fig. 7B). This current was partially (75%) blocked by 30 nM tetrodotoxin (TTX), although 300 nM TTX blocked it completely (Fig. 7B). This confirmed that Oct4/ATSCs engrafted brain slices expressed voltage-gated Na channels. Voltage-clamp recordings were performed at 70 mV in an extracellular solution containing 3 mM Mg_2_. Traces showed spontaneous slow and fast currents indicating that the transplanted Oct4/ATSCs received synaptic contacts from host cells. This current was 98% blocked by CNQX AP5 solution (Fig. 7B). Engrafted Oct4/ATSCs had efficiently migrated and transdifferentiated into functionally active neurons in the hippocampus that actively promotes the amelioration of the lesion cavity (Fig. 7C).

## Discussion

Pluripotency factors, including stemness genes, provide fundamental mechanisms underlying the properties of stem cells. Several factor mediated molecular pathways also engage in cross-talk with one another in order to maintain or obtain stem cell pluripotency. It has been shown that Oct4, Sox2 and Nanog co-regulate certain genes encoding components of signal pathways controlling stem cell behaviors such as cell proliferation or differentiation. They also actively regulate chromatin remodeling via histone modifying complexes, such as MYS3 and SET [19]. According to several recent studies, the pluripotency factor genes are subjected to epigenetic regulation, cell growth and differentiation behaviors via epigenetic reprogramming. Several genes are related to epigenetic regulation, including Jmjd1a and Jmjd2c which are downstream targets of Oct4 [20], [21].

As a member of the POU transcription factor family, Oct4 contains a bipartite DNA binding domain consisting of two subdomains connected by a flexible linker of varying length. Recently, it has been reported that Oct4 activates fibroblast growth factor 4 (FGF4), osteopontin adhesion molecule, transcription coactivator Utf1, and transcription factor Rex1. An enhancer element of the FGF4 gene is activated by Oct4 in cooperation with the high mobility group box transcription factor Sox2 [22]–[25]. The mechanisms involved in the Oct4 regulation in ES have been widely investigated, but the adult stage (including postnatal and fetus stages) of Oct4 function has not been identified even though it had recently been associated with the undifferentiated pluripotent state of stem cell populations derived from various adult human tissues or organs such as bone marrow-derived multipotent adult progenitor cells. Additionally, CD133-positive pluripotent progenitors isolated from cord blood have also been found to be an Oct4 positive population, with a significant reduction in expression following endothelial cell differentiation *in vitro* [26].

Recently, RNAi-mediated gene knockdown was used to extend the analysis of Oct4 function to human ATSCs. It was found that Oct4 knockdown in ATSCs led to a decrease in stem cell identity and increased differentiation into a mesodermal lineage, but that also led to a decrease in neural differentiation ability of ATSC cells at the morphological and molecular levels. Additionally, Oct4 knockdown dramatically led to apoptotic cell death of cultured ATSC cells along with inducing the activation of apoptotic genes such as Caspase 3 and 9 and Bax and c-PARP expressions. When Oct4 expression was recovered by exogenic Oct4 gene overexpression in damaged cells, ATSCs recovered to normal and viable cell with cell survival related signal pathway molecule such as Akt/PI3K, Bcl2, and ERK1/2. This demonstrates that Oct4 is essential for ATSC self renewal and survival, and establishes a conserved role in maintaining pluripotency in mammals. Several previous studies have shown that even tissue specific somatic cells can de-differentiate into progenitor cells capable of acquiring different functions such as pluripotency. In our study, Oct4/ATSCs overexpress not only Oct4, Sox-2, Nanog, and Rex1, but also c-Myc to obtain active self-renewal activity with pluripotency after exogenic Oct4 transfection. On the other hand, Oct4/ATSC exhibited prominent p21 and p53 gene downregulation. Our results showed that Oct4/ATSC can increase developmental potential following reprogramming via the overexpression of the embryonic transcription factors, Rex1, Oct4, and Oct4-dependent Nanog and Sox2. Most notably, Oct4/ATSC reprogrammed somatic nuclei to express the POU family member Rex1 via DNA demethylation. Thus, the components of pluripotent ATSCs have the potential to elicit reprogramming events in a somatic genome. The proliferation of Oct4/ATSCs is promoted significantly by supplementation of exogenic Oct4 with highly improved transdifferentiation activity. The results of these studies indicate that ATSCs possess their own multipotency to reprogram into more primitive stem cells, with the exception of chromosomal abnormalities and point mutations (data not showed). Thus, the exposure of Oct4/ATSCs may provide a good *in vitro* model to demonstrate the mechanisms of re-differentiation from Oct4/ATSC, which would provide insight into the molecular mechanisms of ATSC proliferation. Although the ERK MAPKs generally regulate cell growth and differentiation, and the JNK and p38 family MAPKs preferentially mediate stress, there is now an increasing amount of evidence to suggest that the activation of the ERK/MAPKs can also be stimulated by a variety of stress stimuli [14], [27]–[29]. Our recent study indicated that exogenic Oct4 can activate MEK and ERK1/2 following the induction of de-differentiation. Such a change was also detected with Akt activation. This study showed for the first time that transcription factor Oct4 could induce a reversible change of ATSCs to a more immature reprogrammed state, via the PI3K/Akt-mediated pathway and JAK/STAT3-mediated signals (Figure S1). The proliferation-associated signal pathway with a high level of TERT activity and conserved telomere length occurring in Oct4/ATSCs and their gene expression revealed a reversion toward a more immature phenotype. These results provided some insight into the manner in which human ATSC gene expression responds to exogenic Oct4 treatment.

In our study, the introduction of exogenic Oct4 to autologous ATSCs provides a simple method for the production of primitive stem cells via enhanced growth activity and multipotency, and may also be utilized in the investigation of the mechanisms underlying differentiation and cell reprogramming. As an *in vivo*, traumatic brain injury model, Oct4/ATSC has dramatic regenerative ability with improved function. With the highly improved growth activity, differentiation potency also specified by the de-differentiation processes of adult stem cells, and the relative ease with which these multipotent stem cells can be obtained, there is potentially a large reservoir of therapeutic stem cells for use in improved cell-based disease therapies.

## Materials and Methods

### Culture of human adipose tissue-derived stem cells

Human raw fat tissue obtained from the patient abdomen (as following patient's approval document) was processed according to established methodologies to determine stem cell vascular function [30]. This work was approved by Seoul National University Institutional Review Board (IRB No. 0603/001-002-07C1) and the ethics committee specifically approved that procedure. To isolate stromal cells, the samples were digested at 37°C for 30 min with 0.075% collagenase TypeIV. Enzyme activity was neutralized with α-modified Eagle's medium (α-MEM), containing 10% fetal bovine serum (FBS) and centrifuged at 1,200×*g* for 10 min. Medium was replaced first at 48 hrs and then every 4th day thereafter. Cell viability was evaluated via visual cell counts in conjunction with trypan blue exclusion. In all viability assays, triplicate wells were used for each condition, and each experiment was repeated at least three times. Raw data from each experiment were analyzed using analysis of variance with Fisher or *t* test.

### 5-Bromo-2′-deoxyuridine incorporation

The proliferation activity of cultured ATSC cells was measured by their uptake of 5-bromo-2′-deoxyuridine (BrdU) using a commercial kit (BD Pharmingen). Briefly, cells were incubated for 24 h in growth media supplemented with 10 µM BrdU (Sigma, Deisenhofen, Germany). In 24 h and 48 h after Oct4 transfection (5–8 ug/ml), ATSC cells were fixed using 4% paraformaldehyde for 15 minutes and then immunostained using an anti-BrdU antibody according to the manufacturer's instructions. Fluorescence was monitoring using an inverse confocal laser scanning microscope (LSM 5, Pascal, Carl Zeiss, Jena, Germany). Relative cell proliferation was calculated from the fluorescence of 5 fields of view (n = 4 for each condition). Differences in relative cell proliferation were assessed by two-way ANOVA followed by a post-hoc *t*-test. Differences between two conditions at *P*≤0.05 and *P*<0.001 was considered statistically significant.

### Non-radioisotopic Telomerase Assay

Telomerase activity was assessed using a modified telomeric repeat amplification protocol (TRAP) assay, in accordance with the manufacturer's instructions (BD Science). Protein extracts were prepared from the ATSC controls and the de-ATSC. Protein extracts (0.5 µg) prepared from each cell line was incubated in the presence of synthetic oligonucleotide (telomerase-specific primer, 5′-AATCCGTCGAGCAGAG TT-3′), which could be the substrate for the addition of telomeric repeats by telomerase. If telomerase activity was detected in the extracts, the oligonucleotide was elongated and could function as a template in subsequent polymerase chain reactions (PCR). PCR was conducted in the presence of nucleotides, and the formation of the amplification products was assessed via the monitoring of telomerase repeat amplification. PCR reaction products were separated on 12.5% non-denaturing acrylamide gels and stained using Syber-Gold dye (Molecular Probes, Eugene, OR, USA). Quantification of telomerase for comparisons with telomerase activity in the Oct4 transfected ATSC and the ATSC controls was conducted via the PCR enzyme-linked immunosorbent assay procedure suggested by the manufacturer (BD Science).

### Small interfering RNA inhibition experiment

For Knock-down experiment against Oct4 synthesized siRNA duplex was obtained from DHARMACON. For transient transfection, about 60% confluent cells in 6-wells were transfected with 10 µM siRNA using Lipofectamine (Invirogen). Cells were allowed to stabilize for 48 h before being used in experiment. Cells were harvested after 24 hours for RNA isolation. Silencer Negative Control siRNA (catalog number 4611; Ambion, Inc.) was utilized as a control for non-specific gene silencing. The transfection of siRNAs was conducted using DharmaFECT siRNA transfection reagents, in accordance with the manufacturer's instructions (Dharmacon RNA Technologies). Two complementary hairpin siRNA template oligonucleotides harboring the 21 nt target sequences of the human Rex1 were employed for transient transfection using 50 nM siRNA. Furthermore, the quantity of siRNA was optimized in accordance with the manufacturer's instructions. Three separate Rex1 siRNAs (Silencer® predesigned siRNAs; Ambion) and scrambled siRNAs with the same nucleotide content were assessed. When compared with unrelated control siRNAs and scrambled siRNAs, the Rex1 siRNAs resulted in an 80–90% reduction in Rex1 mRNA levels, as determined via real-time PCR. The siRNA that provided the most efficient inhibition (90–95%) was utilized for the experiments. The inhibition of ATSC growth was detected by transfection of Oct4 siRNA into ATSCs cells and counted dye-exclusive viable cells for 6 days.

### TUNEL Assay

The death of apoptotic cells after H_2_O_2_ treatment was estimated in terms of their ability to reduce the dye MTT (3, 4, 5-dimethyl thiazol-2-yl) -2, 5-diphenyl tetrazolium bromide (Sigma) to blue purple formazan crystal. The effect of H_2_O_2_ on the induction of cell death was determined with the TdT in situ apoptosis detector kit (Roche, USA), used according to the manufacturer's specifications. After fixation, cells with 4% paraformaldehyde were incubated in TUNEL reaction mixture containing deoxynucleotidyl transferase (TdT) buffer with TdT and biotinylated dUTP, incubated in a humid atmosphere at 37°C for 90 min, and then washed with PBS. The cells were incubated at room temperature for 30 min with anti-horseradish peroxidase-conjugated antibody, and the signals were visualized with diaminobenzidine. The results were analyzed using a Fluorescence Microscope (Leica Microsystem, PA). TUNEL-positive apoptotic cells were quantified by counting of positively stained cells. Three digital microscopic images at a magnification of 100× were randomly captured at the areas where the positive cells and the number of positively stained cells in the three images were averaged.

### Bisulfite modification and sequencing of genomic DNA

Firstly, isolation and purification of Genomic DNA was performed through phenol/chloroform extraction and ethanol-precipitation processes. Bisulfite conversion was conducted using the EZ DNA Methylation–Gold Kit (Zymo Research, USA), as indicated by the manufacturer. Briefly, unmethylated cytosines in DNA were converted into uracil via the heat-denaturation of DNA and with a specifically designed CT conversion reagent. DNA was then desulphonated and subsequently cleaned and eluted. The bisulfite-modified DNA was then immediately utilized for PCR or stored at or below −20°C. The PCR reactions were conducted in a MyGenie 96 Gradient Thermal Block (Bioneer, Daejeon, South Korea) in accordance with the following protocol: 95°C for 15 min, 40 cycles of 95°C for 20 sec, 43–58°C for 40 sec, 72°C for 30 sec, followed by an extension at 72°C for 10 min, and soaking at 4°C. Following electrophoresis on a 1.5% agarose gel, the remaining PCR products were cloned into bacteria (DH5α) by a pGEM T-Easy Vector System I (Promega, Madison, WI, USA). DNA extracted from bacterial clones was analyzed via sequencing with the M13 reverse primer (5′-AGCGGATAACAATTTCACACAGGA-3′), using an ABI 3730XL capillary DNA sequencer (Applied Biosystems, Foster City, CA, USA) and represented as rows of circles, with each circle symbolizing the methylation state of one CpG [31].

### Evaluation of trans-differentiation properties of Oct4 overexpressed cells in vitro and in vivo BT mouse model

For the induction of neural differentiation, we cultured neurospheres in a neurobasal medium (NB; Invitrogen, Gaithersburg, MD, USA), supplemented with B27 (Invitrogen), 20 ng/ml of bFGF, and 10 ng/ml of EGF (Sigma) for 4–7 days. The culture density of the spheroid bodies was maintained at 20–50 cells/cm^2^ to prevent self-aggregation. Then, neurospheres derived from the cells were layered and cultured further on PDL-laminin double-coated well plates. To determine the expression of the neural markers, differentiated ATSC were fixed in 4% paraformaldehyde (PFA) fixative solution for 30 minutes at room temperature. After extensive washing in PBS, the cells were blocked for 30 minutes at room temperature with 1% normal goat serum. The cells were then incubated with primary antibodies against anti-TuJ1 (1∶500; Sigma) and anti-GFAP (1∶1500; DAKO, USA). After extensive washing, the cells were incubated with FITC or Texas-Red conjugated secondary antibodies (1∶250; Molecular Probes, USA). We then analyzed the cells via fluorescence microscopy (Leica Microsystems, PA, USA) [32].

### Electrophysiological Recording

Electrophysiological evaluation (evoked action potential of engrafted Oct4/ATSCs was performed before, immediately after and 30 days after the sciatic taxonomy and Oct4/ATSCs transplantation. Under anesthesia, the rat's left sciatic nerve and fourth digital nerve were exposed. Bipolar hooked platinum recording and stimulating electrodes were used to induce and record electrical activity. The stimulating electrode was placed under the proximal sciatic nerve and the recording electrode was placed under the fourth digital nerve. The evoked action potential in responding to the stimuli (one ms, 500 mV) in the ipsilateral sciatic nerve was recorded using Powerlab-800 system (AD Instruments, Colorado Springs, CO, http://www.adinstrumentsinc.com).

### Oligonucleotide microarray and data analysis

Samples for gene array analysis were prepared from the total RNA, and microarray analysis was conducted in accordance with the manufacturer's recommendations. Fragmented cRNA (15 mg) was hybridized for 16 hours at 45°C to the Affymetrix HG-U95A array for the comparison study (Affymetrix, Santa Clara, CA, USA). After hybridization, the probe arrays were scanned at 3 mm resolution using the Genechip System confocal scanner. The output from the microarray analysis was merged with the Unigene or Genebank descriptor and stored as an Excel (Microsoft Corp., Redmond, WA, USA) data spreadsheet. The definition of a 2-fold change, or no change in the expression of individual genes was predicated on the ranking of the difference call (DC) from two comparisons (2′1), namely, no change (NC) of expression for individual genes was merged with the Unigene or GeneBank descriptor and stored as an Excel data spreadsheet. The reproducibility of paired experiments was evaluated on the basis of the coefficient of variation (CV; SD/mean) for fold change (FC). The CV of FC must be ≤1.0. Finally, genes with a FC>2.0 were considered to be significant. These cut-off values represented a conservative estimate of the numbers of genes whose expression levels differed between samples. Gene categorization was based on a literature review. All microarray data in this study was described in accordance with MIAME guidelines. And the resulting data from DNA sequencing has been deposited in GeneBank and include the accession numbers.

### miRNA analysis

The miRNA assay probes correspond to 470 well annotated human miRNA sequences (miRBase: http://microrna.sanger.ac.uk/, version 9.1, February 2007 Release) and 265 miRNAs identified recently. To maximize assay specificity, candidate probes were examined collectively to minimize sequence similarity between probes, particularlyat their 3′-ends. Briefly, 15 ul of the cDNA synthesis reaction was added to 5 ul of the multiplexed MSO pool (MAP, Illumina) and 30 ul of a reagent containing streptavidin paramagnetic particles (OB1, Illumina), heated to 70°C, and allowed to anneal to 40°C. All 735 human miRNAs were assayed simultaneously. After binding and washing, the annealed MSOs were extended through the cDNA primer, forming an amplifiable product. The extended oligos were eluted from the streptavidin beads and added to a PCR reaction, in which one of the universal primers was fluorescently labelled and the other universal primer was biotinylated. The PCR products were captured on streptavidin paramagnetic beads, washed and denatured to yield single stranded fluorescent molecules to hybridize to the arrays. The universal arrays used for fluorescent reporting consist of capture oligos immobilized on beads and randomly assembled into wells etched in sentrix universal bead chip (1536 bead array). The identity of each bead is determined before hybridization to the miRNA assay product, and the same arrays are used to report the results of similar assays employing the address sequence technique (GoldenGate_ Genotyping Assay, DASL Gene Expression Assay, GoldenGate Methylation Assay). Arrays were scanned on the BeadArray Reader, and automatic image registration and intensity extraction software was used to derive intensity data per bead type corresponding to each miRNA. All microarray data in this study was described in accordance with MIAME guidelines. And the resulting data from DNA sequencing has been deposited in GeneBank and include the accession numbers.

### Statistical Analysis

All data were expressed as the means±SEM from five or more independent experiments. The statistical significance of difference between groups was calculated using Student's two tailed *t*-test.

## Supporting Information

Figure S1 Potential signal pathway activated in Oct4/ATSCs
(0.06 MB PDF)

Table S1 Functional Classification of downregulating genes by exogenic OCT4 transfection.
(0.30 MB DOC)
