# Supplementary figures and images for: Regulation of Adipose Tissue Stromal Cells Behaviors by Endogenic Oct4 Expression Control
Source: PLoS One. 2009 Sep 24;4(9):e7166. doi: 10.1371/journal.pone.0007166 (PMC2747014; doi:10.1371/journal.pone.0007166)

Supplementary Fig.1

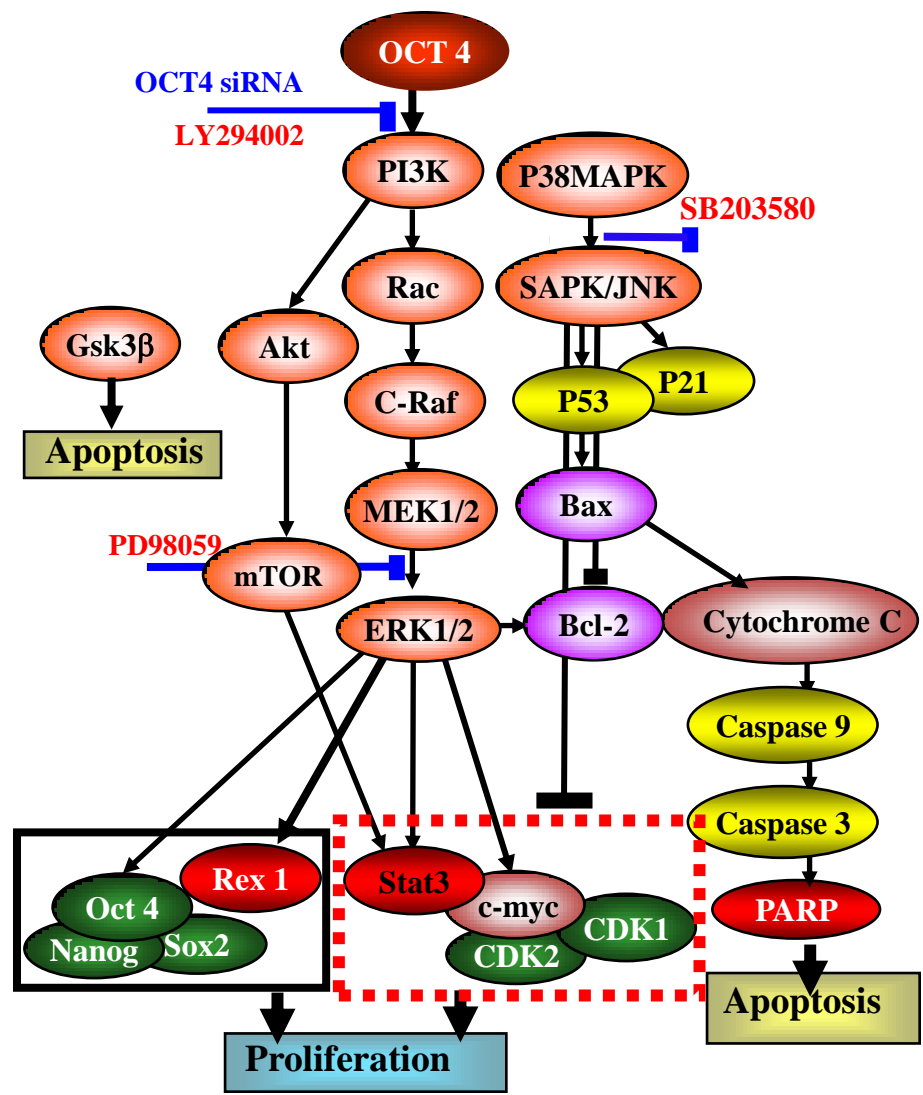

Supplement: Figure S1 — Potential signal pathway activated in Oct4/ATSCs (0.06 MB PDF) [file pone.0007166.s001.pdf]
