# Supplementary material for: Regulation of Adipose Tissue Stromal Cells Behaviors by Endogenic Oct4 Expression Control
Source: PLoS One. 2009 Sep 24;4(9):e7166. doi: 10.1371/journal.pone.0007166 (PMC2747014; doi:10.1371/journal.pone.0007166)
Supplement: Table S1 — Functional Classification of downregulating genes by exogenic OCT4 transfection. (0.30 MB DOC) [file pone.0007166.s002.doc]

**Table S1. Functional Classification of downregulating genes by exogenic OCT4 transfection.**

| Apoptosis | |  |
| --- | --- | --- |
| NM_004655 | AXIN2 | axin 2 (AXIN2) |
| NM_152872 | FAS | Fas |
| NM_207585 | IFNAR2 | interferon receptor 2 |
| NM_001561 | TNFRSF9 | tumor necrosis factor receptor superfamily 9 |
| **Carbohydrate metabolism** | | |
| NM_175907 | ZADH2 | zinc binding alcohol dehydrogenase, domain containing 2 |
| NM_152783 | D2HGDH | D-2-hydroxyglutarate dehydrogenase |
| NM_021615 | CHST6 | carbohydrate (N-acetylglucosamine 6-O) sulfotransferase 6 |
| NM_005110 | GFPT2 | glutamine-fructose-6-phosphate transaminase 2 |
| NM_153282 | HYAL1 | hyaluronoglucosaminidase 1 |
| NM_015892 | GALNAC | B cell RAG associated protein (GALNAC4S-6ST) |
| NM_002168 | IDH2 | isocitrate dehydrogenase 2 |
| **Cell adhesion** | |  |
| NM_006675 | TSPAN9 | tetraspanin 9. |
| NM_016533 | NINJ2 | ninjurin 2 |
| NM_018209 | ARFGAP1 | ADP-ribosylation factor GTPase activating protein 1 |
| NM_020872 | CNTN3 | contactin 3 |
| NM_002593 | PCOLCE | procollagen C-endopeptidase enhancer |
| NM_019555 | ARHGEF3 | Rho guanine nucleotide exchange factor |
| NM_002589 | PCDH7 | protocadherin 7 |
| NM_130830 | LRRC15 | leucine rich repeat containing 15. |
| NM_000090 | COL3A1 | collagen, type III, alpha 1 |
| NM_014021 | SSX2IP | synovial sarcoma, X breakpoint 2 interacting protein |
| NM_002404 | MFAP4 | microfibrillar-associated protein 4 |
| NM_177556 | TRO | trophinin (TRO) |
| NM_016174 | CERCAM | cerebral endothelial cell adhesion molecule |
| NM_015419 | MXRA5 | matrix-remodelling associated 5 |
| NM_020962 | NOPE | neighbor of Punc E11 |
| NM_007046 | EMILIN1 | elastin microfibril interfacer 1 |
| NM_021219 | JAM2 | junctional adhesion molecule 2 |
| NM_003637 | ITGA10 | integrin, alpha 10 |
| **Cell cycle control and Proliferation** | | |
| NM_001759 | CCND2 | cyclin D2 |
| NM_130435 | PTPRE | protein tyrosine phosphatase, receptor type E |
| NM_033260 | FOXQ1 | forkhead box Q1 |
| NM_005461 | MAFB | v-maf musculoaponeurotic fibrosarcoma oncogene homolog |
| NM_001613 | ACTA2 | actin, alpha 2, smooth muscle, aorta |
| NM_014970 | KIFAP3 | kinesin-associated protein 3 |
| NM_004444 | EPHB4 | EPH receptor B4 |
| NM_016084 | RASD1 | RAS, dexamethasone-induced 1 |
| NM_152322 | BTBD11 | BTB (POZ) domain containing 11 |
| NM_002581 | PAPPA | pregnancy-associated plasma protein A |
| NM_032457 | PCDH7 | protocadherin 7 (PCDH7) |
| NM_022910 | NDRG4 | NDRG family member 4 |
| NM_003239 | TGFB3 | transforming growth factor, beta 3 |
| NM_177947 | ARMCX3 | armadillo repeat containing, X-linked 3 |
| **Cell migration** | |  |
| NM_002609 | PDGFRB | platelet-derived growth factor receptor, beta polypeptide |
| NM_003633 | ENC1 | ectodermal-neural cortex1 |
| NM_005786 | TSHZ1 | teashirt zinc finger homeobox 1 |
| NM_018212 | ENAH | enabled homolog |
| NM_130435 | PTPRE | protein tyrosine phosphatase, receptor type, E |
| NM_002658 | PLAU | plasminogen activator, urokinase |
| NM_173485 | TSHZ2 | teashirt zinc finger homeobox 2 |
| NM_152882 | PTK7 | PTK7 protein tyrosine kinase 7 |
| NM_153026 | PRICKLE1 | prickle homolog 1 |
| NM_001511 | CXCL1 | chemokine (C-X-C motif) ligand 1 |
| NM_016081 | PALLD | palladin, cytoskeletal associated protein |
| NM_198252 | GSN | gelsolin |
| NM_001613 | ACTA2 | actin, alpha 2, smooth muscle, aorta |
| NM_003174 | SVIL | supervillin (SVIL) |
| NM_00100533 | DNM1 | dynamin 1 |
| **Development** | |  |
| NM_018530 | GSDMB | gasdermin B |
| NM_005902 | SMAD3 | SMAD family member 3 |
| NM_003392 | WNT5A | wingless-type MMTV integration site family, member 5A |
| NM_006617 | NES | nestin |
| NM_002775 | HTRA1 | HtrA serine peptidase 1 |
| NM_005224 | ARID3A | AT rich interactive domain 3A |
| NM_003250 | THRA | thyroid hormone receptor, alpha |
| NM_005786 | TSHZ1 | teashirt zinc finger homeobox 1 |
| NM_017637 | BNC2 | basonuclin 2 |
| NM_020872 | CNTN3 | contactin 3 |
| NM_002589 | PCDH7 | protocadherin 7 |
| NM_003633 | ENC1 | ectodermal-neural cortex 1 |
| NM_020795 | NLGN2 | neuroligin 2 |
| NM_012445 | SPON2 | spondin 2 |
| NM_001406 | EFNB3 | ephrin-B3 |
| NM_000168 | GLI3 | GLI-Kruppel family member GLI3 |
| NM_033260 | FOXQ1 | forkhead box Q1 |
| NM_001878 | CRABP2 | cellular retinoic acid binding protein 2 |
| NM_003062 | SLIT3 | slit homolog 3 |
| NM_002160 | TNC | tenascin C |
| NM_015441 | OLFML2B | olfactomedin-like 2B |
| **Antioxidation and free radical removal** | | |
| NM_000104 | CYP1B1 | cytochrome P450, family 1, subfamily B, polypeptide 1 |
| NM_001561 | TNFRSF9 | tumor necrosis factor receptor superfamily, member 9 |
| NM_130830 | LRRC15 | leucine rich repeat containing 15 |
| NM_015892 | GALNAC6ST | B cell RAG associated protein |
| NM_000584 | IL8 | interleukin 8 |
| NM_000600 | IL6 | interleukin 6 |
| NM_003944 | SELENBP1 | selenium binding protein 1 |
| NM_00102484 | CD82 | CD82 molecule |
| NM_015696 | GPX7 | glutathione peroxidase 7 |
| NM_022572 | PNKD | paroxysmal nonkinesiogenic dyskinesia |
| NM_007112 | THBS3 | thrombospondin 3 |
| NM_002658 | PLAU | plasminogen activator, urokinase |
| NM_139125 | MASP1 | mannan-binding lectin serine peptidase 1 |
| NM_138455 | CTHRC1 | collagen triple helix repeat containing 1 |
| NM_002982 | CCL2 | chemokine (C-C motif) ligand 2 |
| NM_000120 | EPHX1 | epoxide hydrolase 1, microsomal (xenobiotic) |
| NM_000849 | GSTM3 | glutathione S-transferase M3 |
| NM_00102519 | CES1 | carboxylesterase 1 |
| NM_001511 | CXCL1 | chemokine (C-X-C motif) ligand 1 |
| NM_207585 | IFNAR2 | interferon (alpha, beta and omega) receptor 2 |
| NM_004120 | GBP2 | guanylate binding protein 2, interferon-inducible |
| NM_019554 | S100A4 | S100 calcium binding protein A4 |
| NM_130386 | COLEC12 | collectin sub-family member 12 |
| **Chromatin packaging and remodeling** | | |
| NM_00101739 | SFRS14 | splicing factor, arginine/serine-rich 14 |
| NM_005224 | ARID3A | AT rich interactive domain 3A |
| NM_001387 | DPYSL3 | dihydropyrimidinase-like 3 |
| NM_139247 | ADCY4 | adenylate cyclase 4 |
| NM_003199 | TCF4 | transcription factor 4 |
| NM_005786 | TSHZ1 | teashirt zinc finger homeobox 1 |
| NM_00103350 | CSTF3 | cleavage stimulation factor 3 |
| **Signal transduction** | | |
| NM_004460 | FAP | fibroblast activation protein, alpha |
| NM_001235 | SERPINH1 | serpin peptidase inhibitor, clade H, member 1 |
| NM_002775 | HTRA1 | HtrA serine peptidase 1 |
| NM_014746 | RNF144 | ring finger protein 144 |
| NM_139125 | MASP1 | mannan-binding lectin serine peptidase 1 |
| NM_000396 | CTSK | cathepsin K |
| NM_002581 | PAPPA | pregnancy-associated plasma protein A |
| NM_002658 | PLAU | plasminogen activator, urokinase |
| NM_004994 | MMP9 | matrix metallopeptidase 9 |
| NM_001129 | AEBP1 | AE binding protein 1 |
| NM_019609 | CPXM1 | carboxypeptidase X (M14 family), member 1 |
| NM_178006 | STARD13 | START domain containing 13 |
| NM_000165 | GJA1 | gap junction protein, alpha 1 |
| NM_002023 | FMOD | fibromodulin (FMOD) |
| NM_002345 | LUM | lumican (LUM) |
| NM_006329 | FBLN5 | fibulin 5 (FBLN5) |
| NM_133503 | DCN | decorin (DCN) |
| NM_130435 | PTPRE | protein tyrosine phosphatase, receptor type, E |
| NM_006486 | FBLN1 | fibulin 1 |
| NM_001406 | EFNB3 | ephrin-B3 |
| NM_014262 | LEPREL2 | leprecan-like 2 |
| NM_00101799 | SH3PXD2B | SH3 and PX domains 2B |
| NM_002658 | PLAU | plasminogen activator, urokinase |
| NM_032457 | PCDH7 | protocadherin 7 |
| NM_152882 | PTK7 | PTK7 protein tyrosine kinase 7 |
| NM_015419 | MXRA5 | matrix-remodelling associated 5 |
| NM_001878 | CRABP2 | cellular retinoic acid binding protein 2 |
| NM_003882 | WISP1 | WNT1 inducible signaling pathway protein 1 |
| NM_001996 | FBLN1 | fibulin 1 |
| NM_00102484 | CD82 | CD82 molecule |
| NM_003013 | SFRP2 | secreted frizzled-related protein 2 |
| NM_004460 | FAP | fibroblast activation protein |
| NM_005902 | SMAD3 | SMAD family member 3 |
| NM_004444 | EPHB4 | EPH receptor B4 |
| NM_207585 | IFNAR2 | interferon (alpha, beta and omega) receptor 2 |
| NM_002923 | RGS2 | regulator of G-protein signalling 2 |
| NM_021905 | GABBR1 | gamma-aminobutyric acid (GABA) B receptor, 1 |
| NM_033135 | PDGFD | platelet derived growth factor D |
| NM_173843 | IL1RN | interleukin 1 receptor antagonist |
| NM_006129 | BMP1 | bone morphogenetic protein 1 |
| NM_003239 | TGFB3 | transforming growth factor, beta 3 |
| NM_130435 | PTPRE | protein tyrosine phosphatase, receptor type, E |
| NM_001406 | EFNB3 | ephrin-B3 |
| NM_152882 | PTK7 | PTK7 protein tyrosine kinase 7 |
| NM_023940 | RASL11B | RAS-like, family 11, member B |
| NM_020190 | OLFML3 | olfactomedin-like 3 |
| NM_003485 | GPR68 | G protein-coupled receptor 68 |
| NM_002982 | CCL2 | chemokine (C-C motif) ligand 2 |
| NM_001511 | CXCL1 | chemokine (C-X-C motif) ligand 1 |
| NM_005824 | LRRC17 | leucine rich repeat containing 17 |
| NM_015441 | OLFML2B | olfactomedin-like 2B |
| NM_00104043 | MAPK8IP3 | mitogen-activated protein kinase 8 interacting protein 3 |
| NM_016084 | RASD1 | RAS, dexamethasone-induced 1 |
| NM_021643 | TRIB2 | tribbles homolog 2 |
| NM_023009 | MARCKSL1 | MARCKS-like 1 |
| NM_177403 | RAB7B | RAB7B, member RAS oncogene family |
| **Ion transport** | |  |
| NM_004172 | SLC1A3 | solute carrier family 1member 3 |
| NM_012281 | KCND2 | potassium voltage-gated channel, Shal-related member 2 |
| NM_005502 | ABCA1 | ATP-binding cassette, sub-family A (ABC1), member 1 |
| NM_001878 | CRABP2 | cellular retinoic acid binding protein 2 |
| NM_182676 | PLTP | phospholipid transfer protein |

**Table 1. Functional Classification of overexpressing genes after exogenic OCT4 transfection.**

| Metabolism | |  |
| --- | --- | --- |
| NM_021154.3 | PSAT1 | phosphoserine aminotransferase 1 |
| NM_000071.1 | CBS | cystathionine-beta-synthase |
| NM_133436.1 | ASNS | asparagine synthetase |
| NM_006623.2 | PHGDH | phosphoglycerate dehydrogenase |
| NM_021100.3 | NFS1 | NFS1 nitrogen fixation 1 homolog |
| NM_004472.2 | FOXD1 | forkhead box D1 |
| NM_001919.2 | DCI | dodecenoyl-Coenzyme A delta isomerase |
| NM_001018073.1 | PCK2 | phosphoenolpyruvate carboxykinase 2 |
| NM_001013251.1 | SLC3A2 | solute carrier family 3 |
| NM_018290.2 | PGM2 | phosphoglucomutase 2 |
| **Apoptosis** |  |  |
| NM_014314.3 | DDX58 | DEAD (Asp-Glu-Ala-Asp) box polypeptide 58 |
| NM_001225.3 | CASP4 | caspase 4, apoptosis-related cysteine peptidase |
| NM_003805.3 | CRADD | CASP2 and RIPK1 domain containing adaptor death domain |
| NM_022121.2 | PERP | PERP, TP53 apoptosis effector |
| NM_005107.2 | ENDOGL1 | endonuclease G-like 1 |
| NM_001040619.1 | ATF3 | activating transcription factor 3 |
| NM_005627.2 | SGK | serum/glucocorticoid regulated kinase |
| NM_001168.2 | BIRC5 | baculoviral IAP repeat-containing 5 |
| **Cell adhesion and migration** | | |
| NM_002204.1 | ITGA3 | integrin, alpha 3 |
| NM_001794.2 | CDH4 | cadherin 4, type 1, R-cadherin |
| NM_002784.2 | PSG9 | pregnancy specific beta-1-glycoprotein 9 |
| NM_078481.2 | CD97 | CD97 molecule (CD97) |
| NM_139029.1 | CD151 | CD151 molecule |
| NM_012121.4 | CDC42EP4 | CDC42 effector protein 4 |
| NM_004099.4 | STOM | stomatin (STOM) |
| NM_153374.1 | LYSMD2 | LysM, putative peptidoglycan-binding domain 2 |
| NM_002840.3 | PTPRF | protein tyrosine phosphatase, receptor type, F |
| NM_001565.2 | CXCL10 | chemokine (C-X-C motif) ligand 10 |
| NM_000459.2 | TEK | TEK tyrosine kinase, endothelial |
| NM_002462.2 | MX1 | myxovirus (influenza virus) resistance 1 |
| NM_005556.3 | KRT7 | keratin 7 |
| NM_003379.3 | VIL2 | villin 2 |
| NM_002840.3 | PTPRF | protein tyrosine phosphatase, receptor type, F |
| **Cell proliferation and differentiation** | | |
| NM_004417.2 | DUSP1 | dual specificity phosphatase 1 |
| NM_001006932.1 | RPS6KA2 | ribosomal protein S6 kinase, 90kDa, polypeptide 2 |
| NM_018438.4 | FBXO6 | F-box protein 6. |
| NM_001237.2 | CCNA2 | cyclin A2 |
| NM_001786.2 | CDC2 | cell division cycle 2, G1 to S and G2 to M. |
| NM_005429.2 | VEGFC | vascular endothelial growth factor C |
| NM_203291.1 | RBBP8 | retinoblastoma binding protein 8 |
| NM_005107.2 | ENDOGL1 | endonuclease G-like 1 |
| NM_002915.3 | RFC3 | replication factor C3 |
| NM_145899.1 | HMGA1 | high mobility group AT-hook 1 |
| NM_012112.4 | TPX2 | TPX2, microtubule-associated, homolog |
| NM_198436.1 | AURKA | aurora kinase A |
| NM_005733.1 | KIF20A | kinesin family member 20A |
| NM_004441.3 | EPHB1 | EPH receptor B1 |
| NM_014310.3 | RASD2 | RASD family, member 2 |
| NM_003641.3 | IFITM1 | interferon induced transmembrane protein 1 |
| NM_012137.2 | DDAH1 | dimethylarginine dimethylaminohydrolase 1 |
| NM_021913.2 | AXL | AXL receptor tyrosine kinase |
| NM_080591.1 | PTGS1 | prostaglandin-endoperoxide synthase 1 |
| NM_012112.4 | TPX2 | TPX2, microtubule-associated, homolog |
| NM_013277.2 | RACGAP1 | Rac GTPase activating protein 1 |
| NM_000963.1 | PTGS2 | prostaglandin-endoperoxide synthase 2 |
| NM_004095.3 | EIF4EBP1 | eukaryotic translation initiation factor 4E binding protein 1 |
| **Development** | |  |
| NM_016441.1 | CRIM1 | cysteine rich transmembrane BMP regulator 1 |
| NM_199511.1 | CCDC80 | coiled-coil domain containing 80 |
| NM_002167.2 | ID3 | inhibitor of DNA binding 3 |
| NM_203418.1 | RCAN1 | regulator of calcineurin 1 |
| NM_004441.3 | EPHB1 | EPH receptor B1 |
| NM_001430.3 | EPAS1 | endothelial PAS domain protein 1 |
| NM_001006932.1 | RPS6KA2 | ribosomal protein S6 kinase, 90kDa |
| NM_203417.1 | RCAN1 | regulator of calcineurin 1 |
| NM_006988.3 | ADAMTS1 | ADAM metallopeptidase with thrombospondin type 1 motif |
| NM_139072.3 | DNER | delta/notch-like EGF repeat containing |
| NM_002840.3 | PTPRF | protein tyrosine phosphatase, receptor type, F |
| NM_004472.2 | FOXD1 | forkhead box D1 |
| NM_013277.2 | RACGAP1 | Rac GTPase activating protein 1 |
| NM_001018056.1 | VLDLR | very low density lipoprotein receptor |
| NM_017631.4 | DDX60 | DEAD (Asp-Glu-Ala-Asp) box polypeptide 60 |
| NM_002546.3 | TNFRSF11B | tumor necrosis factor receptor superfamily, member 11b |
| NM_001006932.1 | RPS6KA2 | ribosomal protein S6 kinase, 90kDa, polypeptide 2 |
| NM_001039348.1 | EFEMP1 | EGF-containing fibulin-like extracellular matrix protein 1 |
| **Antioxidation and free radical removal** | | |
| NM_022872.2 | IFI6 | interferon, alpha-inducible protein 6. |
| NM_000544.3 | TAP2 | transporter 2, ATP-binding cassette, sub-family B |
| NM_144717.2 | IL20RB | interleukin 20 receptor beta |
| NM_021913.2 | AXL | AXL receptor tyrosine kinase |
| NM_016815.2 | GYPC | glycophorin C |
| NM_080591.1 | PTGS1 | prostaglandin-endoperoxide synthase 1 |
| NM_021105.1 | PLSCR1 | phospholipid scramblase 1 |
| NM_078481.2 | CD97 | CD97 molecule (CD97) |
| NM_000574.2 | CD55 | CD55 molecule |
| NM_002985.2 | CCL5 | chemokine (C-C motif) ligand 5 |
| NM_025218.2 | ULBP1 | UL16 binding protein 1 |
| NM_001548.3 | IFIT1 | interferon-induced protein with tetratricopeptide repeats 1 |
| NM_016817.2 | OAS2 | 2'-5'-oligoadenylate synthetase 2 |
| NM_006084.4 | IRF9 | interferon regulatory factor 9 |
| NM_004688.1 | NMI | N-myc (and STAT) interactor |
| NM_002053.1 | GBP1 | guanylate binding protein 1, interferon-inducible |
| NM_005567.2 | LGALS3BP | lectin, galactoside-binding, soluble, 3 binding protein |
| NM_001806.2 | CEBPG | CCAAT/enhancer binding protein |
| NM_025218.2 | ULBP1 | UL16 binding protein 1 |
| NM_002468.3 | MYD88 | myeloid differentiation primary response gene (88) |
| NM_017947.1 | MOCOS | molybdenum cofactor sulfurase |
| NM_024429.1 | PRKAG2 | protein kinase, AMP-activated, gamma 2 |
| NM_003276.1 | TMPO | thymopoietin (TMPO) |
| **Chromatin packaging and remodeling** | | |
| NM_024625.3 | ZC3HAV1 | zinc finger CCCH-type, antiviral 1 |
| NM_001042426.1 | CENPA | centromere protein A |
| NM_005107.2 | ENDOGL1 | endonuclease G-like 1 |
| NM_002915.3 | RFC3 | replication factor C (activator 1)3 |
| NM_203291.1 | RBBP8 | retinoblastoma binding protein 8 |
| NM_004510.2 | SP110 | SP110 nuclear body protein |
| NM_139266.1 | STAT1 | signal transducer and activator of transcription 1 |
| NM_006079.3 | CITED2 | Cbp/p300-interacting transactivator |
| NM_201437.1 | TCEA1 | transcription elongation factor A1 |
| NM_001040619.1 | ATF3 | activating transcription factor 3 |
| NM_001430.3 | EPAS1 | endothelial PAS domain protein 1 |
| NM_001040443.1 | PHF11 | PHD finger protein 11 |
| NM_004235.3 | KLF4 | Kruppel-like factor 4 |
| NM_001806.2 | CEBPG | CCAAT/enhancer binding protein, gamma |
| NM_004089.3 | TSC22D3 | TSC22 domain family, member 3 |
| NM_001040443.1 | PHF11 | PHD finger protein 11 |
| NM_002526.1 | NT5E | 5'-nucleotidase, ecto |
| NM_015840.2 | ADAR | adenosine deaminase, RNA-specific |
| NM_002201.4 | ISG20 | interferon stimulated exonuclease gene 20kDa |
| NM_024956.3 | TMEM62 | transmembrane protein 62 |
| **Signal transduction** | | |
| NM_007069.2 | HRASLS3 | HRAS-like suppressor 3 |
| NM_003567.2 | BCAR3 | breast cancer anti-estrogen resistance 3 |
| NM_001013398.1 | IGFBP3 | insulin-like growth factor binding protein 3 |
| NM_001565.2 | CXCL10 | chemokine (C-X-C motif) ligand 10 |
| NM_002514.2 | NOV | nephroblastoma overexpressed gene |
| NM_001794.2 | CDH4 | cadherin 4, type 1, R-cadherin (retinal) |
| NM_203401.1 | STMN1 | stathmin 1/oncoprotein |
| NM_006988.3 | ADAMTS1 | ADAM metallopeptidase with thrombospondin type 1 |
| NM_002840.3 | PTPRF | protein tyrosine phosphatase, receptor type, F |
| NM_001039348.1 | EFEMP1 | EGF-containing fibulin-like extracellular matrix protein1 |
| NM_000459.2 | TEK | TEK tyrosine kinase, endothelial |
| NM_005613.3 | RGS4 | regulator of G-protein signalling 4 |
| NM_201525.1 | GPR56 | G protein-coupled receptor 56 |
| NM_020152.2 | C21orf7 | chromosome 21 open reading frame 7 |
| NM_003749.2 | IRS2 | insulin receptor substrate 2 |
| NM_024576.3 | OGFRL1 | opioid growth factor receptor-like 1 |
| NM_018841.4 | GNG12 | guanine nucleotide binding protein, gamma 12 |
| NM_001233.3 | CAV2 | caveolin 2 |
| NM_005533.2 | IFI35 | interferon-induced protein 35 |
| NM_004688.1 | NMI | N-myc interactor |
| NM_005627.2 | SGK | serum/glucocorticoid regulated kinase |
| NM_203418.1 | RCAN1 | regulator of calcineurin 1. |
| NM_004417.2 | DUSP1 | dual specificity phosphatase 1 |
| NM_014310.3 | RASD2 | RASD family, member 2 |
| NM_032387.3 | WNK4 | WNK lysine deficient protein kinase 4. |
| NM_002468.3 | MYD88 | myeloid differentiation primary response gene |
| NM_024429.1 | PRKAG2 | protein kinase, AMP-activated, gamma 2 |
| NM_001006932.1 | RPS6KA2 | ribosomal protein S6 kinase, 90kDa, polypeptide 2 |
| NM_203417.1 | RCAN1 | regulator of calcineurin 1 |
| NM_013277.2 | RACGAP1 | Rac GTPase activating protein 1 |
